# Perception of Young European Otolaryngologists toward Transoral Robotic Surgery in Head and Neck Oncology and Surgery

**DOI:** 10.3390/jcm13113055

**Published:** 2024-05-23

**Authors:** Jerome R. Lechien, Abdul-Latif Hamdan, Nicolas Fakhry, Luigi A. Vaira, Giannicola Iannella, Isabelle M. Gengler, Justin Michel, Thomas Radulesco, Marc Remacle, Stephane Hans, Giovanni Cammaroto, Alberto M. Saibene, Miguel Mayo-Yanez, Antonino Maniaci

**Affiliations:** 1 Robotic Study Group of Young Otolaryngologists, International Federation of Otorhinolaryngological Societies (YO-IFOS), 13005 Paris, France; nicolas.fakhry@ap-hm.fr (N.F.); luigi.vaira@gmail.com (L.A.V.); giannicola.iannella@uniroma1.it (G.I.); isabellemarie.gengler@gmail.com (I.M.G.); justin.michel@ap-hm.fr (J.M.); thomas.radulesco@ap-hm.fr (T.R.); prhans.foch@gmail.com (S.H.); giovanni.cammaroto@gmail.com (G.C.); alberto.saibene@gmail.com (A.M.S.); tnmaniaci29@gmail.com (A.M.); 2 Department of Otolaryngology and Head and Neck Surgery, Foch Hospital, Paris Saclay University, 91190 Paris, France; marc.remacle01@gmail.com; 3 Department of Otolaryngology and Head and Neck Surgery, Division of Broncho-Esophagology, EpiCURA Hospital, UMONS Research Institute for Health Sciences and Technology, University of Mons (UMons), 7000 Mons, Belgium; 4 Elsan Polyclinic of Poitiers, 86000 Poitiers, France; 5 Laboratory of Anatomy and Cell Biology, UMONS Research Institute for Health Sciences and Technology, University of Mons (UMONS), Avenue du Champ de Mars, 6, 7000 Mons, Belgium; 6 Department of Otolaryngology-Head and Neck Surgery, American University of Beirut Medical Center, Beirut 1107, Lebanon; ah77@aub.edu.lb; 7 Department of Otolaryngology and Head and Neck Surgery, Aix-Marseille Univ, AP-HM, La Conception Hospital, 13005 Marseille, France; 8 Maxillofacial Surgery Operative Unit, Department of Medicine, Surgery and Pharmacy, University of Sassari, 07100 Sassari, Italy; 9 Department of “Organi di Senso”, University “Sapienza”, 00161 Rome, Italy; 10 Department of Otolaryngology-Head and Neck Surgery, University of Cincinnati Medical Center, Cincinnati, OH 45267, USA; 11 Aix Marseille University, APHM, CNRS, IUSTI, La Conception University Hospital, ENT-HNS Department, 13005 Marseille, France; 12 Department of Otolaryngology, Head and Neck Surgery, Eich Hospital, 1460 Eich, Luxembourg; 13 Department of Otolaryngology-Head and Neck Surgery, Forli Hospital, 47122 Forli, Italy; 14 Otolaryngology Unit, Santi Paolo e Carlo Hospital, Department of Health Sciences, Università degli Studi di Milano, 26900 Milan, Italy; 15 Department of Otorhinolaryngology-Head and Neck Surgery, Hospital San Rafael (HSR), 15006 A Coruña, Spain; 16 Faculty of Medicine and Surgery, University of Enna Kore, 94100 Enna, Italy

**Keywords:** transoral, robotic, otolaryngology, head and neck, surgery, survey, awareness, young

## Abstract

**Background**: To investigate the perception of young European otolaryngologists (OTOs), i.e., head and neck surgeons, toward transoral robotic surgery (TORS). **Methods**: Members of the Young Confederation of European Otorhinolaryngology-Head and Neck Surgery and Young Otolaryngologists of International Federation of Otorhinolaryngological Societies were surveyed about TORS perception and practice. **Results**: The survey was completed by 120 young OTOS (26%). The most important barriers to TORS were robot availability (73%), cost (69%), and lack of training (37%). The participants believed that the main benefits include better surgical filed view (64%), shorter hospital stay (62%), and better postoperative outcomes (61%) than the conventional approach. Head and neck surgeons considered cT1-T2 oropharyngeal cancers (94%), resection of base of tongue for sleep apnea (86%), or primary unknown cancer (76%) as the most appropriate indications. A total of 67% of TORS surgeons assessed themselves as adequately trained in TORS. **Conclusions**: Young European OTOs report positive perception, adoption, and knowledge of TORS. The cost-related unavailability and the lack of training or access are reported to be the most important barriers for the spread of TORS.

## 1. Introduction

Transoral robotic surgery (TORS) is a promising surgical approach for some benign and malignant lesions [1]. To date, the Da Vinci robot is used worldwide although it is a late development in head and neck surgery compared to gynecology or urology fields [2,3]. The access to robot in otolaryngology may be difficult according to the cost of the robot, the lack of training, and the knowledge of otolaryngologists (OTOs), i.e., head and neck surgeons, toward indications [4]. However, TORS appears as a promising oncological therapeutic approach in some oropharyngeal and supraglottic malignancies and in thyroid surgery [5,6,7]. Nowadays, there is no study surveying the perception and adoption of TORS by European OTOs, especially head and neck surgeons.

The objective of the present study was to survey perception, adoption, and awareness of young European OTOs toward TORS.

## 2. Methods

The members of the research boards of the Young Otolaryngologists of the International Federation of Otorhinolaryngological Societies (YO-IFOS) and the Young Confederation of Otorhinolaryngological Societies have developed an online survey investigating practice, knowledge, perception, and barriers regarding TORS. The survey was composed of 18 questions investigating demographic information (N = 5); TORS experience and practice (N = 3); training (N = 2); barriers/disadvantages/benefits (N = 2); setting (N = 2) access (N = 1); perception of TORS (N = 1); indications (N = 1); and improvements (N = 1; Appendix A).

Participants were invited to judge the most adequate indications of TORS with a 5-point scale ranging from “no indication” (0) to “perfect indication” (4) in a predefined list of head and neck disorders, such as pharyngeal, laryngeal, and nasopharyngeal malignancies, thyroid surgery, and sleep apnea surgery. An approval from the Institutional Review Board was not required for this study. Given the nature of the study (cross-sectional survey without participant identification), an exception was obtained by the local institutional review board (CHUSTP-2022).

### 2.1. Survey Distribution

SurveyMonkey^®^ (SurveyMonkey Inc., San Mateo, CA, USA) was used to develop the survey. Participants completed the survey only once. The survey was emailed to 467 OTOs of the YO-IFOS/IFOS and y-CEORL-HNS on two occasions. Only the responses of European OTOs were considered. The participants’ locations were identified as well as their center/hospital.

### 2.2. Data Collection

The participant responses were collected anonymously. The incomplete responses were excluded from the analysis. According to the survey item, the responses were reported by the entire cohort (all responders, irrespective of the subspecialty) or by considering two groups of responders: head and neck surgeons (TORS vs. non-TORS surgeons) vs. non-head and neck surgeons (e.g., otologists, rhinologists, pediatrics, general otolaryngologists, and phoniatrics). Head and neck surgeons were defined according to the completion of a fellowship in head and neck surgery or a similar training program during the training period in the country of practice. Young otolaryngologists were practitioners of less than 45 years of age according to the IFOS and CEORL criteria. Statistical analyses were carried out with the Statistical Package for the Social Sciences for Windows (SPSS version 22.0; IBM Corp, Armonk, NY, USA). The differences in response between groups were evaluated using a Kruskal–Wallis test or χ^2^ test regarding data.

## 3. Results

One-hundred and twenty young OTOs completed the survey (26%). There were more males than females in the head and neck surgeon group (Table 1). There was no significant difference in years of experience between head and neck and non-head and neck board-certified surgeons (14.0 ± 13.4 vs. 11.5 ± 7.1). Participants worked in academic hospitals (74%), private offices (7%), or both (19%). Among head and neck surgeons, 28 participants never used TORS. The mean experience years of TORS and non-TORS head and neck surgeons were 15.8 ± 14.9 and 11.6 ± 10.8, respectively (*p* = 0.249).

### 3.1. Robot Access

Eighty-one European OTOs (68%) have limited/no access to TORS. Among them, 68 responders (84%) reported being interested in having better access to TORS. The access outcomes for TORS according to the specialty and practice are reported in Table 2. Sixty-one percent of non-TORS head and neck surgeons were interested in using TORS in their practice, but they had limited access. Overall, 13 participants (9%) stated that they were not interested in using TORS.

### 3.2. Benefit, Barrier, and Overall Perception

The perception of participants about barriers and benefits of TORS are reported in Table 3. The most important barriers to TORS were robot availability (73%), cost (69%), and lack of training (37%). Non-head and neck surgeons believed more frequently that the lack of training is an important barrier compared to head and neck surgeons (47% vs. 28%; *p* = 0.034). The participants believed that the main benefits include a better view of the surgical field (64%), a shorter hospital stay (62%), and better postoperative outcomes (61%) than the conventional approach. The superior view of the surgical field was more frequently considered as an important TORS benefit by head and neck surgeons compared to non-head and neck surgeons (78% vs. 47%, *p* = 0.001). Head and neck surgeons believed more frequently that TORS is associated with a shorter hospital stay and better surgical outcome benefits than non-head and neck surgeons, and, consequently, they advocate TORS to colleagues or encourage them to use TORS (Table 3).

The comparison of barrier, benefit, and opinion outcomes between TORS head and neck and non-TORS head and neck surgeons is available in Appendix B. TORS head and neck surgeons believed to a greater degree than non-TORS head and neck surgeons that TORS is important for the future of minimal invasive surgeries in Europe. TORS and non-TORS head and neck surgeons reported similar perceptions about benefits associated with TORS, while the lack of personal training and the docking time are more frequently considered as important barriers by the non-TORS group compared to the TORS group (Appendix B).

### 3.3. Training, Instruments, and Setting

**Figure 1:**
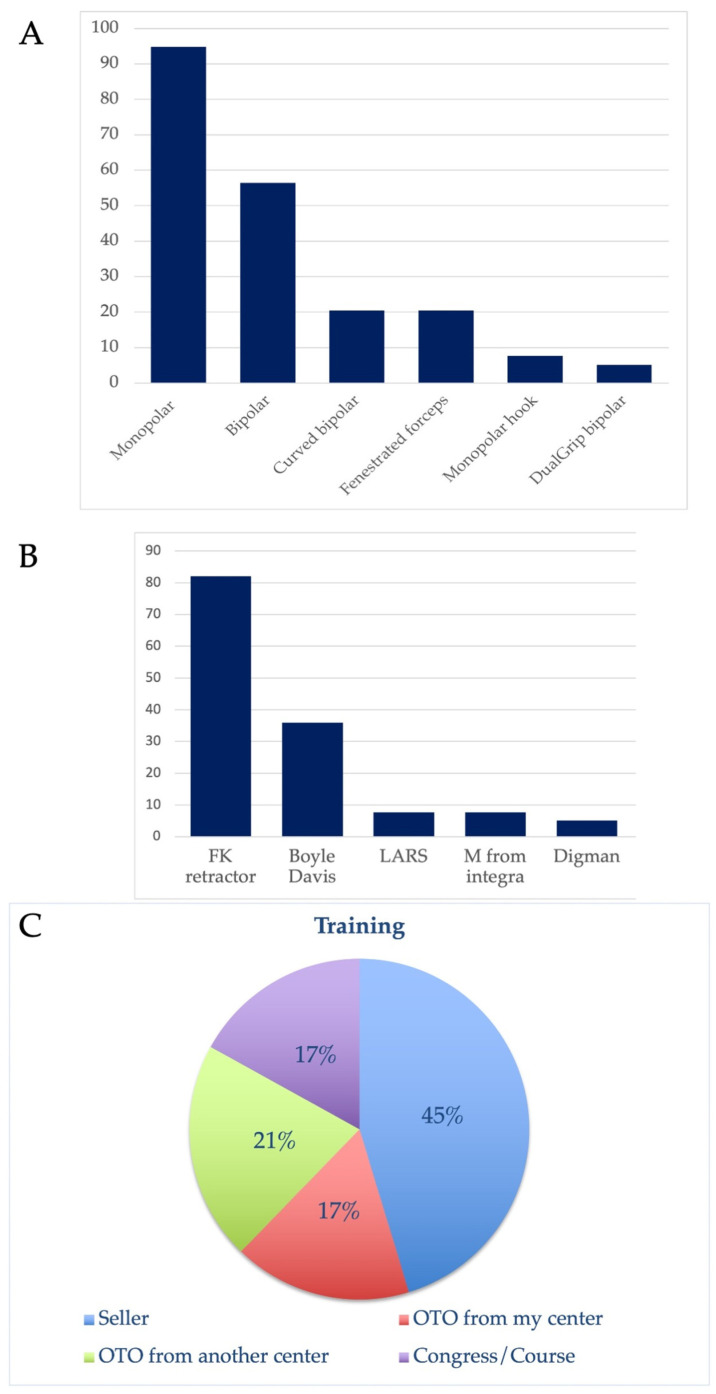
Training and instrument outcomes. The figure included the favorite instruments of TORS surgeons ((**A**): % of use), the mouth retractor used ((**B**); % of use), and the training provided (**C**). Abbreviations: OTO = otolaryngologist.

The training to European TORS head and neck surgeons was provided by the robot seller (61%), experienced colleague(s) of their department (23%) or from another department (28%), or academic/congress course(s) (23%; Figure 1). A total of 26 TORS head and neck surgeons (67%) assessed themselves as adequately trained in TORS. A total of 35.9% TORS head and neck surgeons reported that they received adequate support for their practice by their hospital, while 3.6% non-TORS head and neck surgeons reported the same thing (*p* = 0.002).

European TORS head and neck surgeons carry out an average of 37 procedures yearly. The most used instruments are monopolar spatula (95%), bipolar forceps (56%), curved bipolar (20%), and fenestrated forceps (20%; Figure 1). The mouth retractors used included FK retractor (82%), Boyle Davis (36%), LARS (8%), M from Integra (Moriniere; 8%), and Digman (5%; Figure 1).

### 3.4. Indications of Robotic Surgery

The opinions of head and neck surgeons on TORS indications are reported in Table 4. Head and neck surgeons considered the following conditions as the most appropriate indications for TORS: cT1-T2 oropharyngeal cancers (94%); resection of base of tongue for sleep apnea (86%) or primary unknown cancer (76%); and cT1-T2 hypopharyngeal (73%) and supraglottic (32%) cancers. Head and neck surgeons suggested that the following conditions are contraindications for TORS: cT4 hypopharyngeal cancers (90%); cT3 supraglottic cancers (83%); cT4 oropharyngeal cancers (78%); cT3 hypopharyngeal cancers (73%); cT4 supraglottic cancers (65%); cT1-T2 vocal fold cancers (65%); head and neck dissection (60%); and pharyngeal flap (56%). The rest of the conditions described in Table 4 were not reported clearly as either an indication or a contraindication. The opinions of TORS and non-TORS head and neck surgeons toward the indications of robotic surgery did not differ in most conditions with the exception of cT1-T2 oropharyngeal cancers and partial thyroid surgery. For cT1-T2 oropharyngeal cancers, TORS head and neck surgeons believed more frequently that TORS is an adequate approach than non-TORS head and neck surgeons, while an opposite trend was found for partial thyroid surgery (Table 4). Notably, 53.6% of non-TORS head and neck surgeons stated that they may accept to refer a patient with an adequate indication of TORS to another center, while 21.4% did not agree. The rest of non-TORS head and neck surgeons did not report a strong opinion or skipped the question.

### 3.5. Improvements and Perspectives

Participants considered that the priorities for the future development/improvement of a new generation of robots include the spread of CO_2_ laser (74%); the reduction in the robot arm size and the incorporation of flexible instruments (69%); the integration of imaging–navigation system to guide surgeons in complex resection (51%); the integration of narrow-band imaging with robot camera (51%); and better back strength (33%). According to participants, these improvements are important to enhance the access to hypopharynx (59%); glottis (49%); supraglottic larynx (49%); oropharynx (36%); nasopharynx (31%); and nasal fossae (23%).

## 4. Discussion

Since the first transoral robotic surgery performed in 2006 [8], the number of robotic procedures has substantially increased worldwide. However, the spread of robotic procedures in Europe is currently limited by the cost, the lack of training of the young practitioners, and reimbursements by the healthcare system [9].

In the present investigation, we showed that the young European otolaryngologists and, particularly, head and neck surgeons, reported adequate adoption and perception outcomes toward TORS. This is an important observation because young OTOs represent the future of the workforce in otolaryngology—head and neck surgery. Most head and neck surgeons supported that TORS is associated with a better surgical field view, a shorter hospital stay, and comparable or better postoperative outcomes than those of conventional approaches. The perception of participants corroborates the findings of the literature. Thus, many studies supported that the video endoscope of the Da Vinci robot offers a large, magnified view of confined spaces of most surgical fields in otolaryngology, which may be associated with better operative outcomes than those of conventional approaches [10,11,12]. The TORS-related shorter hospital length and fewer postoperative complication rates were additional perceptions of European OTOs that corroborate the findings of the literature [13,14].

Participants reported that the cost-related robot unavailability and the lack of training remain the primary significant barriers to the use of TORS. Precisely, most TORS head and neck surgeons reported that they did not receive adequate support from their hospital. Surprisingly, only 67% assessed themselves as adequately trained in TORS. In a recent German survey, Mandapathil and Meyer reported that the main reasons for not adopting TORS were costs, lack of interest, and unavailable collaborations with experienced TORS head and neck surgeons [9]. Similar findings were reported by the survey of Krishnan et al. who identified high costs and limited training opportunities and access to and availability of the robotic platform as the most important perceived barriers to TORS in Oceania [15].

In our survey, both non-TORS and TORS head and neck surgeons reported similar overall indication outcomes. Precisely, most head and neck surgeon participants considered sleep apnea-related base of tongue surgery and cT1-T2 oropharyngeal and supraglottic cancers as the most appropriate indications of TORS, which match with the most accepted indications in the literature [6,16,17,18,19]. However, notably, European non-TORS and TORS head and neck surgeons appear to be less familiar with fewer common indications, e.g., Sistrunk procedure, laryngocele excision, nasopharyngeal cancer, or thyroid surgery [20,21,22]. To date, the superiority of TORS over the open procedures for these indications is not demonstrated. Furthermore, the usefulness of TORS in partial or total thyroid surgery is demonstrated [23,24], but these approaches are more frequently carried out in Asia. The adequate knowledge of non-TORS or TORS head and neck surgeons pertaining to TORS indications was similarly observed in the study by Krishnan et al. [15].

Further efforts are needed to develop robotic programs in European academic centers according to the potential advantages of TORS over conventional head and neck surgeries [13,14]. An American study reported that training was associated with a lower rate of positive margins compared to non-robotic surgery [25]. The first step for the implementation of robotic program is the support of the hospital [9], which is an important barrier in Europe according to our participants. Several options may be developed to improve the training of OTOs in Europe, including simulator access, clinical rotations, or surgical courses, which have to be implemented early in a surgeon’s career [26]. The willingness to be trained and the positive perception of European OTOs toward TORS are both important factors that may lead to the further success of such healthcare approaches. Furthermore, participants provided some interesting improvement suggestions for future generations of robot, including the spread of CO_2_ laser in robot, which is already used in few centers, and the reduction in the arm size as in SinglePort Da Vinci robot. These points support the fact that, at baseline, the most widely available robot (Da Vinci robot) was not adapted to otolaryngology surgery. The development of SinglePort and future other models may increase the use in otolaryngology.

The main limitation of this survey was the low participation rate (26%), which may be attributed to the lack of interest in the topic and the poor access to TORS, both making this kind of survey vulnerable to sampling errors and respondent biases. To be precise, it could be conceivable that young practitioners who are not interested in TORS did not respond, which makes it difficult to extrapolate the results. However, the present participation rate was comparable with previous surveys that were conducted in the same young OTO sample (YO-IFOS and y-CEORL-HNS).

## 5. Conclusions

The sample of young European otolaryngologists and head and neck surgeon participants reported positive perception, adoption, and knowledge toward TORS. The cost-related unavailability and the lack of training could be the most important barriers for the spread of TORS. However, these findings need to be confirmed in future large-cohort studies including a more representative sample of otolaryngologists, i.e., head and neck surgeons. The findings of such a survey may help further decisions in increasing TORS interest and awareness in Europe.

## Figures and Tables

**Table 1:** Cohort features.

	Participants	Head and Neck Surgeons	Non-Head and Neck Surgeons	
Outcomes	N = 120	N = 67	N = 53	*p*-Value
Gender (F/M)	40/80	13/54	27/26	0.001
Years of experience (years)	11.6 ± 11.5	14.0 ± 13.4	8.5 ± 7.9	0.018
Main subspecialties				
General otolaryngology	13 (11)	-	13 (24)	-
Head and neck	67 (56)	67	-	
Laryngology	2 (2)	-	2 (4)	
Rhinology	6 (5)	-	6 (11)	
Otology	8 (7)	-	8 (15)	
Pediatrics	10 (8)	-	10 (19)	
Residency	14 (11)	-	14 (26)	
Places of practice				
Academic/university	89 (74)	50 (75)	39 (74)	NS
Private	8 (7)	3 (4)	5 (9)	
Academic and private	23 (19)	14 (21)	9 (17)	

The results are reported as the number of responders (%). Statistical analyses were carried out considering the head and neck and non-head and neck practitioners. Abbreviations: F/M = female/male; NS = non-significant.

**Table 2:** Robot access outcomes of European otolaryngologist, i.e., head and neck surgeons.

	Non-Head and Neck Surgeons (N = 53)	Head and Neck Surgeons (N = 67)		
Access Outcomes		TORS (N = 39)	Non-TORS (N = 28)	*p*-Value
No/limited access and not interested	9 (17)	0 (0)	2 (7)	0.001
No/limited access but interested	33 (62)	7 (18)	17 (61)	0.001
Adequate access but I do not use TORS	11 (21)	0 (0)	8 (29)	0.001
Adequate access but cost-prohibitive	0 (0)	2 (5)	1 (3)	0.001
Adequate access and I use it	0 (0)	30 (77)	0 (0)	0.001

The results are reported as the number of responders (%) considering non-head and neck versus head and neck groups, and, in head and neck group, TORS versus non-TORS practitioners. Abbreviations: N = number; TORS = transoral robotic surgery.

**Table 3:** Perceptions, barriers, and benefits of TORS according to participants.

Overall Opinion	Non-Head and Neck Surgeons (53)	Head and Neck Surgeons (67)	*p*-Value
TORS is associated with many surgical and hospital stay benefits	28 (43)	47 (70)	0.040
There are more disadvantages to TORS than advantages	2 (4)	5 (8)	NS
I trust in TORS for the future	23 (43)	35 (52)	NS
I advocate TORS to my colleagues	5 (9)	15 (22)	0.048
I encourage colleagues to use TORS in the future	9 (17)	24 (36)	0.022
TORS has affected me positively since adoption	3 (6)	19 (28)	0.001
TORS is important for the future of minimal invasive surgeries	23 (43)	36 (54)	NS
Main barriers of TORS			
Robot availability	41 (77)	46 (69)	NS
Cost related to TORS in my healthcare system	41 (77)	42 (63)	NS
Time restraint	14 (26)	13 (19)	NS
Low volumes of procedures performed at my center	16 (30)	16 (24)	NS
Low theoretical volumes of procedures performed with TORS	14 (26)	27 (40)	NS
Lack of personal training	25 (47)	19 (28)	0.034
Lack of interest	8 (15)	4 (6)	NS
Docking time (setting the robot)	9 (17)	8 (12)	NS
Difficulty of exposure of the surgical field	9 (17)	19 (28)	NS
Main benefits			
1. Esthetic benefit (scar)	25 (47)	31 (46)	NS
2. Avoid tracheotomy in some selected cases	28 (53)	40 (60)	NS
3. Shorter hospital stay	32 (60)	42 (63)	NS
4. Better patient postoperative quality of life than in the conventional approach	30 (57)	43 (64)	NS
5. Better view of the operative field than in the conventional approach	25 (47)	52 (78)	0.001
6. Better movements of the robot arm in the operative field than in the conventional approach	25 (47)	41 (61)	NS

The results are reported as the number of responders (%). Abbreviations: NS = non-significant; TORS = transoral robotic surgery.

**Table 4:** Indications of TORS according to practitioners.

	TORS Surgeons					Non-TORS Surgeons					
Indications	0	1	2	3	4	0	1	2	3	4	*p*-Value
Oropharynx											
cT1-T2 oropharyngeal cancer	0	0	0	23.1	76.9	3.6	3.6	3.6	50.0	39.2	0.022
cT3 oropharyngeal cancer	0	17.9	38.5	35.9	7.7	3.8	26.9	30.8	30.8	7.7	NS
cT4a oropharyngeal cancer	42.1	39.5	5.3	13.2	0	48.1	25.9	22.2	3.7	0	NS
Base of tongue											
Sleep apnea syndrome	0	17.9	30.8	51.3	0	0	7.4	33.3	59.3	0	NS
Unknown primary cancer	2.6	7.7	20.5	30.8	38.5	0	3.6	10.7	35.7	50	NS
Larynx											
cT1-T2 supraglottic cancer	0	2.6	5.1	28.2	64.1	0	10.7	7.1	50.0	32.1	NS
cT3 supraglottic cancer	5.1	33.3	30.8	30.8	0	11.1	37.0	18.5	22.2	11.1	NS
cT4a supraglottic cancer	51.3	38.5	10.3	0	0	42.3	30.8	19.2	7.7	0	NS
Total laryngectomy	25.6	30.8	30.8	10.3	2.6	55.6	22.2	14.8	7.4	0	NS
cT1-T2 vocal fold cancer	0	2.6	20.5	43.6	33.3	3.6	3.6	25.0	57.1	10.7	NS
Hypopharynx											
cT1-T2 hypopharyngeal cancer	10.3	61.5	23.1	5.1	0	22.2	51.9	11.1	11.1	3.7	NS
cT3 hypopharyngeal cancer	60.5	36.8	2.6	0	0	55.6	25.9	18.5	0	0	NS
cT4a hypopharyngeal cancer	28.2	35.9	28.2	7.7	0	37.0	29.6	22.2	7.4	3.7	NS
Others											
Nasopharyngeal cancer	20.5	30.8	30.8	15.4	2.6	19.2	19.2	46.2	11.5	3.8	NS
Neck dissection	10.3	43.6	30.8	15.4	0	14.8	55.6	18.5	11.1	0	NS
Partial thyroidectomy (lobectomy)	12.8	12.8	41.0	23.1	10.3	11.1	40.7	14.8	29.6	3.7	0.41
Total thyroidectomy	15.4	17.9	35.9	28.2	2.6	19.2	38.5	11.5	26.9	3.8	NS
Branchial cyst	10.3	30.8	41.0	15.4	2.6	22.2	40.7	18.5	14.8	3.7	NS
Pharyngeal flap	5.1	15.4	41.0	30.8	7.7	11.1	22.2	40.7	25.9	0	NS

The numbers in the table consist of the % of surgeons who rated the indication as perfect (4), good (3), 2 (neutral), 1 (not good), or 0 (contraindication). Abbreviations: NS = non-significant; TORS = transoral robotic surgery.

## Data Availability

Data are contained within the article.

## Appendix A. Survey

1. **Country of practice:………………..**
2. **Center of practice:**
3. **Gender: ………………..**
4. **Place of practice:** Academic–Private (selection of one or both)

**Subspecialty training, if any:**

Laryngology	Head and Neck
Pediatric Otolaryngology	Rhinology
Otology	Residency

5. **Do you have any experience with TORS?** yes–no (never)
6. **Number of years of practice after the end of the residency.**
7. **Number of procedures performed yearly.**
  - … cases
8. **About my opinion/awareness about TORS:**
  - I never used TORS but I am interested to use it.
  - There are many benefits to TORS.
  - There are more disadvantages to TORS than advantages.
  - I trust TORS for the future.
  - I advocate TORS to my colleagues.
  - I encourage my colleagues to adopt TORS.
  - TORS has affected me positively since adoption.
  - TORS has affected me negatively since adoption.
  - The adoption of TORS by my colleagues has affected me positively.
  - The lack of adoption of TORS by my colleagues has affected me negatively.
  - I believe that TORS is the future of minimal invasive surgeries in otolaryngology—head neck surgery.
9. **About the barriers to use TORS:**
  - Robot availability and cost
  - Cost related to TORS in my healthcare system
  - Time restraint
  - Low volumes of procedures performed at my center
  - Low theoretical volumes of procedures performed with TORS
  - Lack of personal training
  - Lack of interest
  - Long setting/docking time
  - Difficulty of surgical field exposure
10. **What are the presumed benefits of TORS according to you?**
  - Esthetic benefit (scar)
  - Avoid tracheotomy in some selected cases
  - Shorter hospital stay
  - Better patient postoperative quality of life
  - Better view of the operative field
  - Better movements of the robot arm in the operative field
  - Others:
11. **According to your opinion, in which condition TORS may be adequate? Score between 0 (not indicated) to 5 (perfect indication).**
  - Oropharyngeal cancer cT1-T2
  - Oropharyngeal cancer cT3
  - Oropharyngeal cancer cT4a
  - Base of tongue resection (sleep apnea syndrome)
  - Tongue base mucosectomy (unknown primary tumor)
  - Supraglottic laryngeal cancer cT1-T2
  - Supraglottic laryngeal cancer cT3
  - Supraglottic laryngeal cancer cT4a
  - Total laryngectomy
  - Vocal cord cancer cT1-T2
  - Hypopharyngeal cancer cT1-T2
  - Hypopharyngeal cancer cT3
  - Hypopharyngeal cancer cT4a
  - Nasopharyngeal cancer (cT1-T2-T3)
  - Neck dissection
  - Hemi-thyroid surgery (limited lesion)
  - Total thyroidectomy (limited lesion)
  - Branchial cyst
  - Pharyngeal flap
12. **About my access to TORS:**
  - I have no access to TORS, and I am not interested to TORS.
  - I have no access to TORS, but I would like to have access.
  - I have adequate access to TORS, but I did not use it.
  - I have adequate access to TORS, and I use it.
  - TORS cases are cost-prohibitive.
13. **Are you ready to refer your patient to a center with a robot for indications where TORS may be performed?** (if you have a robot in your center and if you use it, SKIP this question)
  - Yes
  - No opinion
  - No, I prefer to perform the surgery myself (open or endoscopic surgery).
  - No
14. FOR A SURGEON WHO USES TORS, **about the training:**
  - I received adequate training for TORS.
  - I received good support from my management.
  - My organization (hospital) encourages me to perform TORS.
  - My organization does not encourage me to perform TORS.
15. FOR A SURGEON WHO USES TORS, **my training was organized by:**
  - Training program by seller
  - Senior otolaryngologists from my department
  - Senior otolaryngologists from another department
  - University course/congress
16. FOR A SURGEON WHO USES TORS, **what are the instruments that you use? (skip if you do not use robotic surgery)**
  - Monopolar spatula
  - Maryland bipolar forceps
  - Monopolar hook
  - Curved bipolar (dissector)
  - Fenestrated bipolar forceps
  - Bipolar with dual grip
  - Other: ….
17. FOR A SURGEON WHO USES TORS, **what are the retraction materials that you use to open the mouth?**
  - FK retractor
  - Boyle Davis retractor
  - LARS
  - Digman
  - Other: ….
18. **What are the improvements that seem important for the future model of the robots?**
  - Better access to oropharynx
  - Better access to supraglottis larynx
  - Better access to glottis
  - Better access to hypopharynx
  - Better access to nasal fossea
  - Better access to nasopharynx
  - Integration of GPS based on MRI imaging
  - Integration of CO2 or another laser
  - Integration of narrow-banded imaging system
  - Better back strength
  - Flexible instruments
  - Other: …………………..

**Abbreviation:** TORS = transoral robotic surgery.

## Appendix B. Perceptions, Barriers, and Benefits of TORS among TORS and Non-TORS Head and Neck Surgeons

**Overall Opinion**	**TORS (39)**	**Non-TORS (28)**	***p*-Value**
TORS is associated with many surgical and hospital stay benefits	29 (74)	18 (64)	NS
There are more disadvantages to TORS than advantages	1 (3)	4 (14)	NS
I trust TORS for the future	23 (59)	12 (43)	NS
I advocate TORS to my colleagues	13 (33)	2 (7)	0.011
I encourage colleagues to use TORS in the future	19 (49)	5 (18)	0.009
TORS is important for the future of minimal invasive surgeries	27 (69)	9 (32)	0.003
Main barriers of TORS			
Robot availability	29 (74)	17 (61)	NS
Cost related to TORS in my healthcare system	22 (56)	20 (71)	NS
Time restraint	6 (15)	7 (25)	NS
Low volumes of procedures performed at my center	9 (23)	7 (25)	NS
Low theoretical volumes of procedures performed with TORS	17 (44)	10 (36)	NS
Lack of personal training	7 (18)	12 (43)	0.026
Lack of interest	1 (3)	3 (11)	NS
Docking time (setting the robot)	2 (5)	6 (21)	0.042
Difficulty of exposure of the surgical field	13 (33)	6 (21)	NS
Main benefits			
1. Esthetic benefit (scar)	18 (46)	13 (46)	NS
2. Avoid tracheotomy in some selected cases	26 (67)	14 (50)	NS
3. Shorter hospital stay	27 (69)	15 (54)	NS
4. Better patient postoperative quality of life	27 (69)	16 (57)	NS
5. Better view of the operative field	29 (74)	23 (82)	NS
6. Better movements of the robot arm in the operative field	24 (62)	17 (61)	NS
The results are reported as the number of responders (%). Abbreviations: NS = non-significant; TORS = transoral robotic surgery.			
